# Impact of mitochondrial nitrite reductase on hemodynamics and myocardial contractility

**DOI:** 10.1038/s41598-017-11531-3

**Published:** 2017-09-21

**Authors:** Peter Dungel, Carina Penzenstadler, Mostafa Ashmwe, Sergiu Dumitrescu, Tanja Stoegerer, Heinz Redl, Soheyl Bahrami, Andrey V. Kozlov

**Affiliations:** 0000 0001 0723 5126 grid.420022.6 L. Boltzmann Institute for Experimental and Clinical Traumatology in AUVA center, Vienna, Austria

## Abstract

Inorganic nitrite (NO_2_^−^) can be reduced back to nitric oxide (NO) by several heme proteins called nitrite reductases (NR) which affect both the vascular tonus and hemodynamics. The objective of this study was to clarify the impact of several NRs on the regulation of hemodynamics, for which hemodynamic parameters such as heart rate, blood pressure, arterial stiffness, peripheral resistance and myocardial contractility were characterized by pulse wave analysis. We have demonstrated that NO_2_^−^ reduced to NO in RBCs predominantly influences the heart rate, while myoglobin (Mb) and mitochondria-derived NO regulates arterial stiffness, peripheral resistance and myocardial contractility. Using *ex vivo* on-line NO-detection, we showed that Mb is the strongest NR occurring in heart, which operates sufficiently only at very low oxygen levels. In contrast, mitochondrial NR operates under both hypoxia and normoxia. Additional experiments with cardiomyocytes suggested that only mitochondria-derived generation of NO regulates cGMP levels mediating the contractility of cardiomyocytes. Our data suggest that a network of NRs is involved in NO_2_^−^ mediated regulation of hemodynamics. Oxygen tension and hematocrit define the activity of specific NRs.

## Introduction

Nitric oxide (NO) is a key player in the regulation of hemodynamic parameters such as peripheral resistance^1^, arterial pressure^2,3^, aortic stiffness, myocardial contractility^4,5^, heart rate, cardiac index^6,7^, and as a consequence tissue perfusion^8^. Various isoforms of NO synthase (NOS) are the main sources of NO under normoxic conditions. However, NOS become inefficient under hypoxic conditions^9^, due to the fact that this enzyme family is oxygen dependent^10^. Nitrite, the second major source of NO, becomes relevant exclusively under hypoxic conditions. Hypoxia has been shown to facilitate the reduction of nitrite to NO, a process catalyzed by so called nitrite reductases (NR) complementary to oxygen-dependent NO synthases^11^. For this reason, nitrite administration has already been suggested for therapeutic treatment of diseases associated with impaired oxygen delivery^12,13^. Both dietary nitrate and nitrate have also been shown to regulate blood pressure^14,15^. Several reports suggest that hemodynamic effects of nitrite occur not only under hypoxic but also under normoxic conditions. In healthy volunteers under normoxic conditions circulating nitrite concentrations have been shown to correlate with systolic and diastolic blood pressure^16,17^ and the administration of nitrite resulted in the dilation of conduit arteries^18^. These data suggest that nitrite-mediated NO release may not only contribute to improve hemodynamics under hypoxic/ischemic conditions but also to physiological regulation of hemodynamics. There are three major sources of nitrite in the body (i) oxidation of NO formed by different NOS, (ii) absorption from food and (iii) the release from specific drugs, such as nitroglycerin. Hobbs *et al*. showed that beetroot bread, containing large amount of nitrate, which is reduced to nitrite in the gastrointestinal tract, increased endothelium-independent vasodilation and decreased diastolic blood pressure and arterial stiffness in healthy men^19^.

Several groups reported that diverse NRs regulate hemodynamics, from which two were found in red blood cells (RBC). Gladwin and co-authors suggested hemoglobin (Hb) in RBC as a key regulator of hemodynamics^20,21^, while Ahluwalia and coworkers presented evidence that blood pressure is regulated predominantly by xanthine oxidoreductase (XOR) which is also localized in RBCs^22^. In contrast Rassaf’s group reported that Mb located in blood vessels, not Hb or XOR, is the major NR responsible for hemodynamic effects of nitrite^23,24^. In addition, Zweier and coauthors suggested that cytoglobin is the key NR responsible for nitrite effects in aorta ring assays^25^. In our previous studies we established the mitochondrial electron transport chain as a potent NR^26^, in particular, in cardiomyocytes^27^. Since, there is such controversial discussion with regard to the individual contribution of all these NRs to the regulation of hemodynamics, the objectives of this study were to develop appropriate experimental models to study the contribution of different NRs in both blood and tissue and to clarify whether there is one major NR or a network of NRs regulating hemodynamics, and to define hemodynamic parameters predominantly regulated by single NRs.

## Results

**Figure 1:**
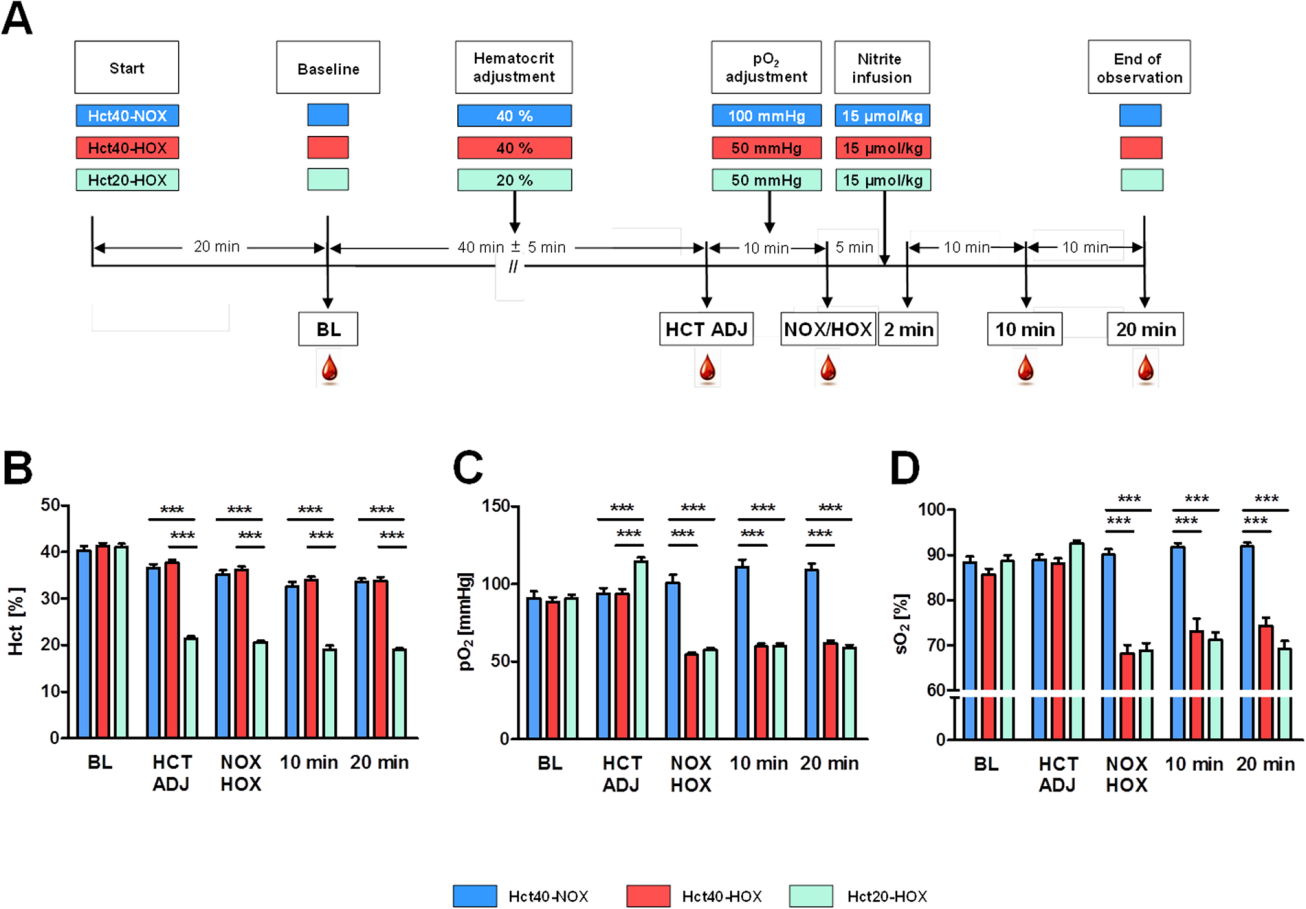
(**A**–**D**) Experimental design and measurement of hematocrit (Hct), arterial oxygen partial pressure (pO_2_) and arterial oxygen saturation (sO_2_) over time. *In vivo* experiments were performed in three groups: with normal Hct under (a) normoxic (Hct40-NOX: blue bars) and (b) hypoxic (Hct40-HOX: red bars) conditions and (c) halved Hct under hypoxic (Hct20-HOX: green bars) conditions. Experimental design includes anesthesia and catheter placement for Hct adjustment (HCT ADJ), pO_2_ adjustment (NOX: normoxic group 100 mmHg; HOX: hypoxic groups: 50 mmHg), hemodynamic monitoring and blood sampling. A bolus of nitrite (15 µmol/kg) was infused intra venously after pO_2_ adjustment. Blood samples were taken at baseline (BL), after HCT ADJ, after NOX/HOX adjustment, 10 and 20 minutes after nitrite infusion (**A**). Changes on Hct (**B**), pO_2_ (**C**) and sO_2_ (**D**) values over time in all three groups determined by blood gas analysis. Data are expressed as mean ± SEM of at least n = 8, ***p < 0.001. One animal died upon adjustment of Hct.

*In vivo* experiments to investigate the hemodynamic effects of nitrite were performed in three groups of rats (Fig. 1A). Two of these groups had a normal hematocrit (Hct) value (40%) and treatment occurred under both normoxic (NOX) (group#1; Hct40-NOX-group) and hypoxic (HOX) (group#2; Hct40-HOX-group) conditions. To clarify the contribution of blood Hb/RBCs under hypoxic conditions, we decreased the Hct in a third group to 20% (group#3; Hct20-HOX-group). In all groups the Hct decreased marginally in the course of the experiments due to continuous infusion of anesthetics and blood withdrawal followed by fluid resuscitations (Fig. 1B). Hct adjustment resulted in an increase in arterial partial pressure (pO_2_) in the Hct20-HOX group. The induction of hypoxia resulted in a drop of pO_2_ of approximately 50% (42% Hct40-HOX vs 50% Hct20-HOX) and a decrease in Hb saturation (sO_2_) of approximately 25% (23% Hct40-HOX vs 25% Hct20-HOX, respectively) in both hypoxic groups (Fig. 1C,D). A decrease in Hct did not influence Hb saturation. The values of pH were identical in all three groups (data not shown).

**Figure 2:**
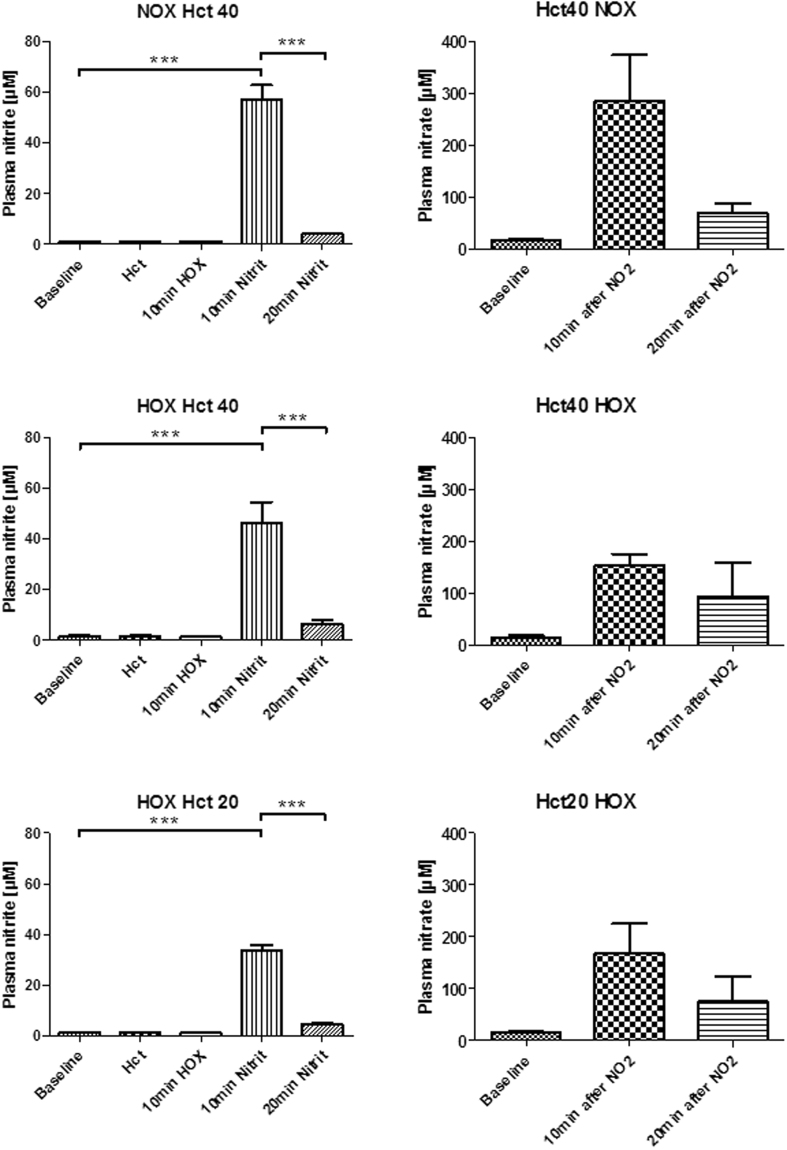
Plasma levels of nitritre (left) and nitrate (right). Both nitrite and nitrate peaked 10 min after a bolus injection of 15 µmol/kg sodium nitrite and were markedly decreased after 10 min.

A bolus injection of 15 µmol/kg sodium nitrite led to a drastic increase of both nitrite and nitrate plasma levels. There was a significant difference in plasma nitrite levels after 10 min between NOX40 and HOX20. Nitrate and nitrate levels peaked 10 min after nitrite injection but substantially decreased already after 20 min and the decrease was more pronounced for nitrite compared to nitrate (Fig. 2).

**Figure 3:**
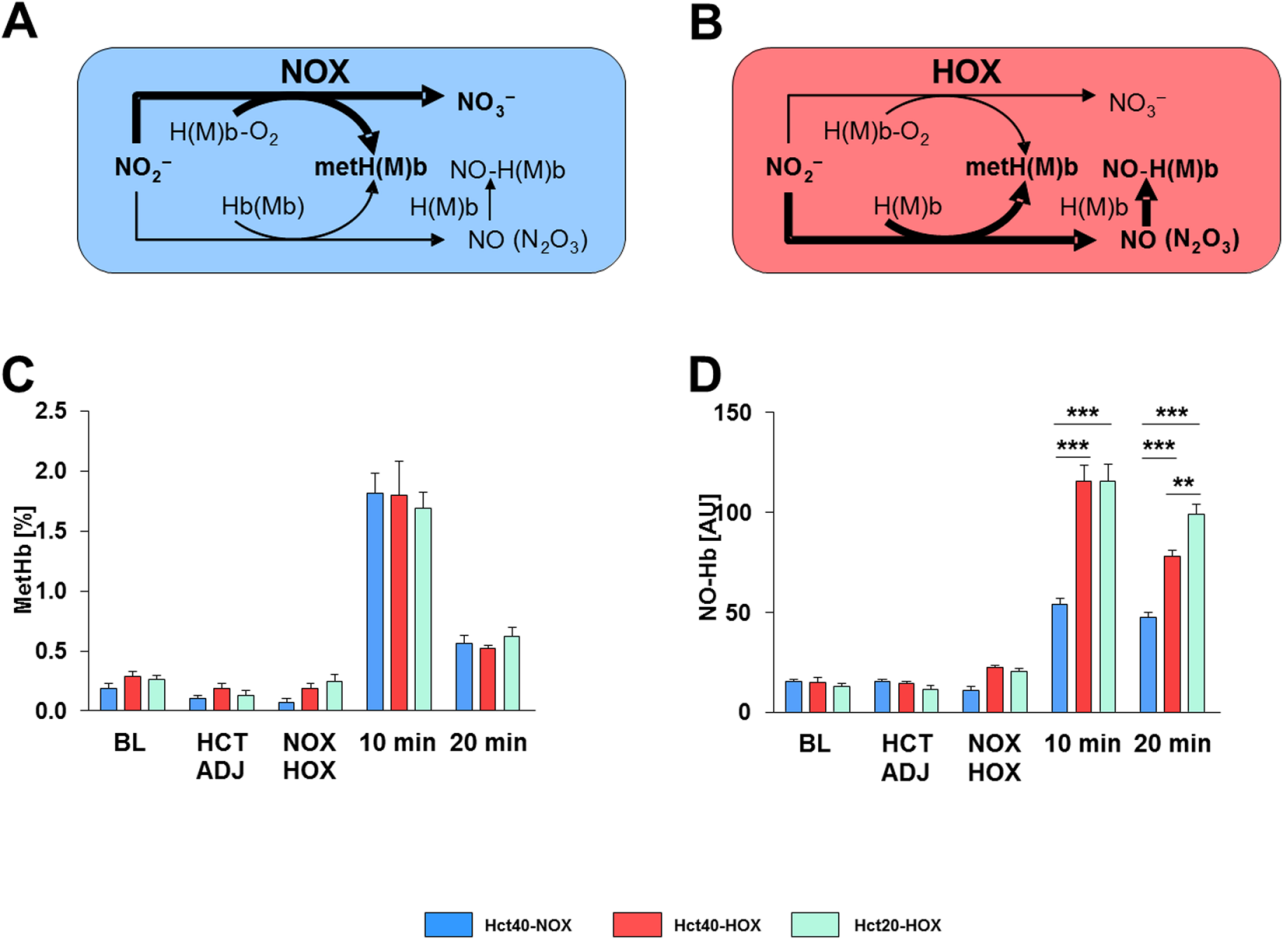
(**A**–**D**) Effect of nitrite infusion on the formation of methemoglobin (metHb) and nitric oxide hemoglobin (NO-Hb) in red blood cells (RBCs). Schematic illustration of nitrite metabolism in oxygenated RBCs; the predominant pathway is the formation of biologically inactive NO_3_^−^ and metHb (**A**). Schematic illustration of nitrite metabolism in deoxygenated RBCs; the predominant pathway is the formation of NO-Hb complexes and metHb (**B**). Nitrite mediated formation of metHb (**C**) determined by EPR and NO-Hb (**D**) over time in RBCs determined by electron paramagnetic resonance spectroscopy in Hct40-NOX (blue bars), Hct40-HOX (red bars) and Hct20-HOX (green bars) group. Blood samples were taken at baseline (BL), after HCT ADJ, after NOX/HOX, 10 and 20 minutes after nitrite infusion. Data are expressed as mean ± SEM of at least n = 8, **p < 0.01, ***p < 0.001. One animal died upon adjustment of Hct.

A schematic view of the interaction of nitrite and Hb in dependence of oxygen is shown in Fig. 3. Under normoxic conditions (Fig. 3A), the predominant pathway of nitrite is the oxidation to nitrate, while under hypoxia (Fig. 3B) nitrite is preferentially reduced to NO, which in turn reacts with Hb to form NO-Hb complexes. Both reactions yield methemoglobin (metHb). This is in line with our experimental data showing no difference in metHb levels between the groups (Fig. 3C). In comparison, the levels of NO-Hb were remarkably higher in both HOX groups and after 20 minutes the levels of NO-Hb in Hct20-HOX group were significantly higher compared to the Hct40-HOX group (Fig. 3D).

**Figure 4:**
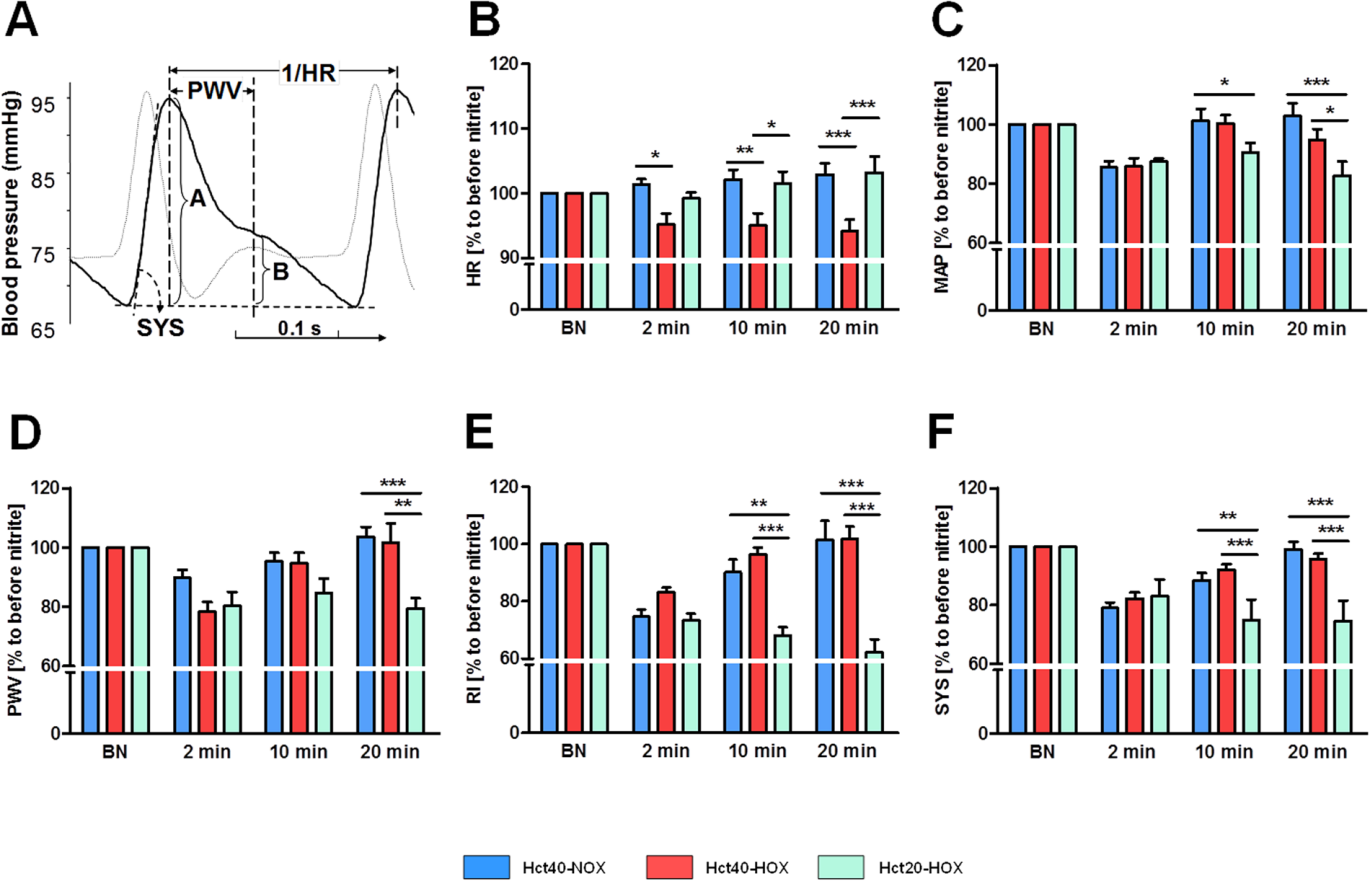
(**A**–**D**) Effect of nitrite infusion on hemodynamic parameters examined by pulse wave analysis. Illustration and calculation of pulse wave analysis: heart rate (HR), mean arterial blood pressure (MAP), pulse wave velocity (PWV), indicating aorta stiffness and systolic slope (SYS), indicating myocardial contractility. Reflection index (RI) was calculated by dividing B/A, indicating peripheral resistance. Solid line represents a typical pulse wave; dotted line is the first derivative from pulse wave identifying the diastolic phase (**A**). Effect of nitrite infusion on the hemodynamic parameters HR (**B**), MAP (**C**), aorta stiffness (**D**), myocardial contractility (**E**) and peripheral resistance (**F**) in Hct40-NOX (blue bars), Hct40-HOX (red bars) and Hct20-HOX (green bars) group. Pulse waves were analysed before nitrite infusion (BN) and after 2, 10 and 20 minutes of observation. Data are expressed as mean ± SEM of n = 9, *p < 0.05, **p < 0.01, ***p < 0.001.

To investigate the effects of nitrite infusion on different hemodynamic parameters we monitored blood pressure and examined the alteration of the shape of pulse waves. Figure 4A depicts a typical pulse wave and the determination of the heart rate (HR), mean arterial blood pressure (MAP), pulse wave velocity (PWV) characterizing aorta stiffness, reflection index (RI), characterizing peripheral resistance and finally the systolic slope (SYS), characterizing myocardial contractility. Figure 4B–F show the hemodynamic changes in heart rate, MAP, aorta stiffness, peripheral resistance and myocardial contractility, respectively.

Nitrite infusion under normoxic conditions (Hct40-NOX) did not influence the heart rate but caused a temporary decrease in MAP, aorta stiffness, peripheral resistance and myocardial contractility, namely a drop after 2 min which recovered after 10 to 20 min (Fig. 4B,C,D,F).

Induction of moderate hypoxia (Hct40-HOX) remarkably and permanently decreased the heart rate (Fig. 4B), which was already significant two minutes after nitrite infusion. The mean values of MAP, aorta stiffness, peripheral resistance and myocardial contractility dropped after nitrite injection (determined after 2 min) but recovered during 20 min to baseline levels (Fig. 4C–F). There was no significant difference between Hct40-HOX and Hct40-NOX groups at any timepoint. On the contrary, a decrease in Hct under hypoxia (Hct20-HOX) abolished the effect of nitrite on heart rate, but facilitated NO-like effects of nitrite on the other hemodynamic parameters, namely a permanent decrease in the values of MAP, aorta stiffness, peripheral resistance and myocardial contractility.

**Figure 5:**
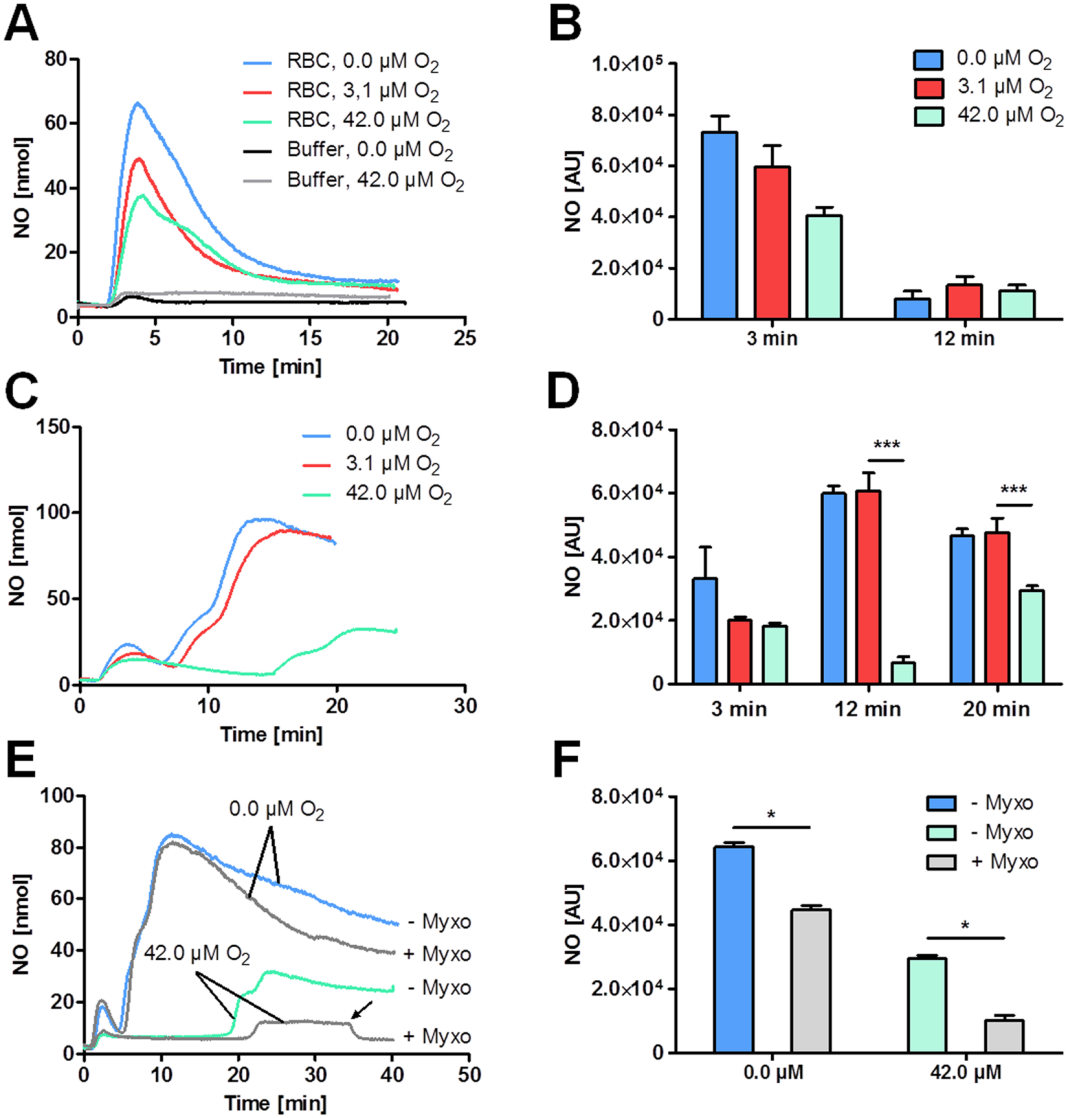
(**A–F**) Effect of different oxygen concentrations on nitrite reduction in red blood cells (RBCs) and heart homogenates determined by NO analysis. Time curve of NO release in RBCs (**A**) and heart homogenates (**C**,**E**) measured at 0.0 µM (blue line), 3.1 µM (red line) and 42.0 µM (green line) oxygen (O_2_) concentration. The contribution of dilution buffer is represented at 0 µM (black line) and 42 µM (grey line) oxygen (O_2_) concentration (**A**). Statistical evaluation of NO release in RBCs (**B**) and heart homogenates (**D**). Mitochondrial contribution to NO release in heart homogenates determined by addition of myxothiazol (Myxo, grey lines), a specific inhibitor of mitochondrial complex III at 0.0 µM and 42 µM O_2_ concentration (**E**,**F**). Arrow indicates addition of potassium cyanide, a specific inhibitor of mitochondrial complex IV. Nitrite was added at 0 min. Data are expressed as mean ± SEM of at least n = 5, *p < 0.05, **p < 0.01, ***p < 0.001.

To further characterize the contribution of NRs located in either tissue or in RBCs/blood, we analyzed nitrite reduction in ex *vivo* experiments. In these experiments we simulated NO release during hypoxia using an on-line chemiluminescence-based NO detection system. The measurements were performed at 0.0 µM, 3.1 µM, and 42 µM O_2_ concentrations. The concentrations of 3.1 and 42 µM O_2_ were selected because they are close to the half-saturation value of oxygen of Mb and Hb, respectively. The addition of nitrite to RBCs resulted in immediate NO release reaching a maximum approx. 2 min after addition of nitrite (Fig. 5A). The increase in oxygen concentration resulted in a decrease in the amount of released NO. NO was not released if nitrite was added to the incubation buffer without RBCs. Figure 5B shows the statistical evaluation of five independent experiments. The addition of nitrite to heart homogenates resulted in a biphasic kinetics of NO release. The first transient peak was again observed approx. 2 min after addition of nitrite (Fig. 5C). This peak was similar to those observed in RBCs. Compared to that, the second NO peak reached its maximum 12 min after nitrite stimulation under 0.0 µM and 3.1 µM O_2_ concentration. The addition of nitrite under 42.0 µM O_2_ resulted in the disappearance of the peak at 12 min and a less intensive peak appeared at approx. 22 min (Fig. 5C). The Fig. 5D shows the statistical evaluation of these results from 5 independent experiments. To clarify the involvement of mitochondria in tissue-located nitrite reduction, we examined the effects of myxothiazol (Myxo) on NO release in heart homogenates at 0.0 µM O_2_ and 42 µM O_2_. Myxothiazol, a specific mitochondrial inhibitor of complex III, partially inhibited the 12 min peak of NO release at 0.0 µM O_2_, (Fig. 5E) and fully inhibited the peak at 22 min occurring at 42 µM O_2_. Potassium cyanide (KCN), a specific inhibitor of mitochondrial complex IV, completely abolished the release of NO. The quantification of these result are presented in Fig. 5F.

The Suppl. Figure 1 shows that the release of NO from nitrite in the presence of heart homogenate is substantially higher than the background NO release from NO_2_^−^ or NO_3_^−^ in the absence of heart homogenate.

To examine the contribution of xanthine oxidoreductase (XOR) in the release of NO we tested effect of allopurinol, known as a specific inhibitor of XOR. The addition of allopurinol completely inhibited NO release suggesting that XOR may be predominantly involved in the release of NO (Suppl. Figure 1). In the following experiment, we tested the NO release from Mb (Suppl. Figure 2). We observed a similarity in the NO release patterns between heart homogenate and myoglobin. Both efficiently released NO at 0 and 3 µM of oxygen, but the release was substantially inhibited by 42 µM O_2_ (Suppl. Figure 2). Interestingly, allopurinol similarly to heart homogenate also nearly completely inhibited the release of NO catalyzed by Mb (Suppl. Figure 2). This suggests that allopurinol inhibits also other than XOR nitrite reductases, such as Mb. Alternatively, it can scavenge NO formed. To test the last assumption we added either allopurinol or saline to NO solutions under anaerobic conditions incubated 5 min and injected into the NO detection chamber. The results are presented in (Suppl. Figure 2); one can see that allopurinol does not affect NO levels, consequently allopurinol does not scavenge NO.

**Figure 6:**
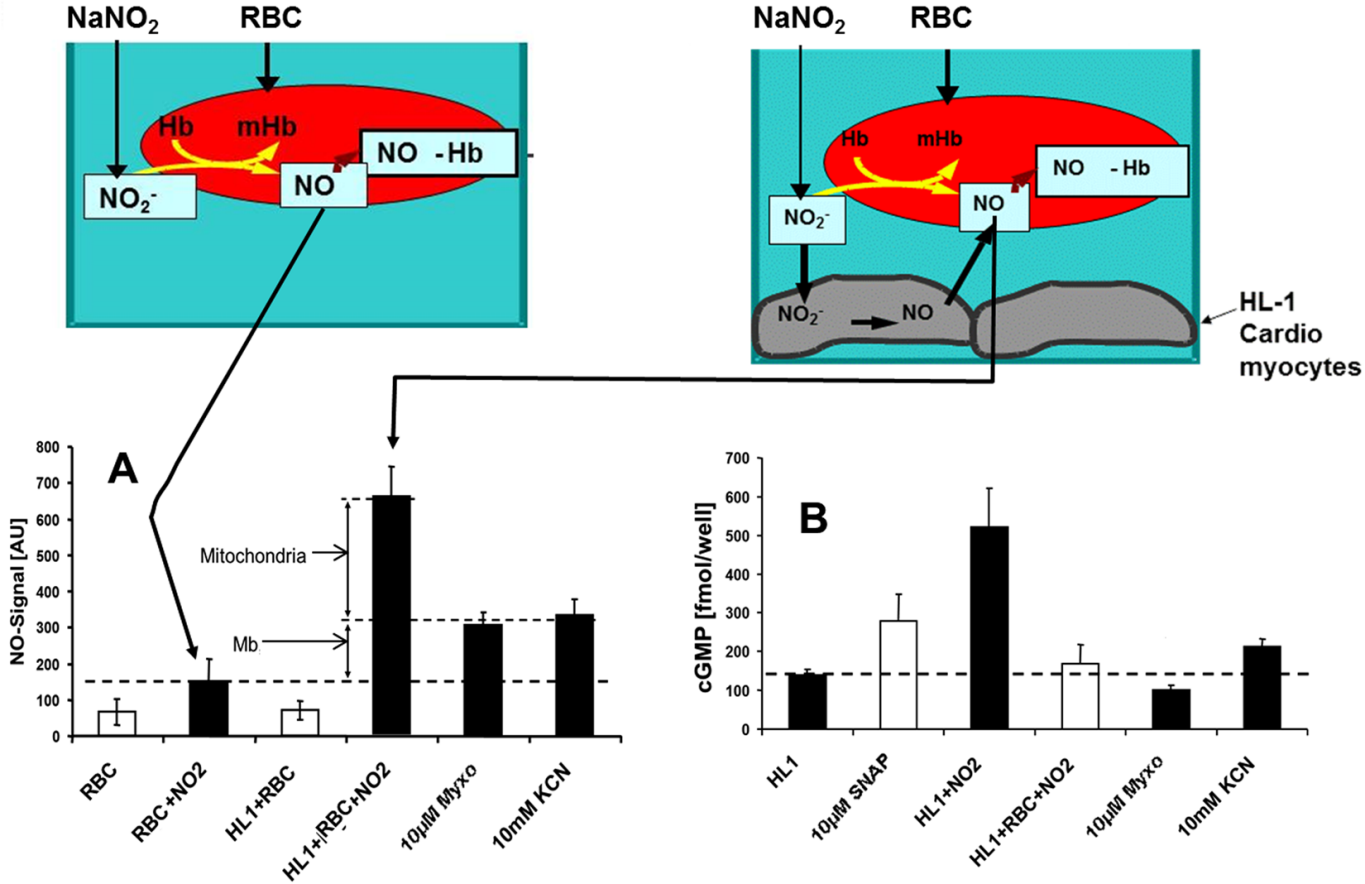
(**A**,**B**) Intracellular effects contributing to hemodynamic parameters assessed by NO-Hb formation and cGMP synthesis in different cell cultures. Contribution of nitrite (30 µM, final concentration), myxodiazol (Myxo) and potassium cyanide (KCN) on NO-Hb formation in cell cultures of red blood cells (RBCs) and cardiomyocyte (HL-1) (**A**). Contribution of SNAP, nitrite, myxodiazol (Myxo) and potassium cyanide (KCN) on cGMP formation in cell cultures of RBCs and cardiomyocyte (HL-1) (**B**). Controls are displayed with open bars. Data are expressed as mean ± SEM of three independent experiments with 4 replicates each, *p < 0.05, ***p < 0.001.

The next set of experiments was performed in a co-culture of RBCs and HL-1 cardiomyocytes. In order to simulate the effect of lowered Hct used in the *in vivo* experiments we also varied the ratio of RBCs to HL-1 cardiomyocytes in the *in vitro* model. The experiments were performed under anaerobic conditions in order to activate all nitrite reductases. The concentration of NO was determined by analysis of NO-Hb complexes by electron paramegnetic resonance spectroscopy (EPR) as it was done in *in vivo* experiments. Addition of nitrite to RBCs alone resulted in the formation of a small amount of NO-Hb via the pathway displayed in the inset (Fig. 6A). In the presence of HL-1 the generation of NO was strongly increased due to the additional generation of NO by the cardiomyocytes. The addition of myxothiazol and KCN resulted in a significant but only partial inhibition of NO generation (Fig. 6A), suggesting that mitochondria only partially contribute to NO release, as it was also shown in the experiments using heart homogenate. In contrast, NO-mediated cGMP synthesis was completely abolished by both myxothiazol and KCN (Fig. 6B).

**Figure 7:**
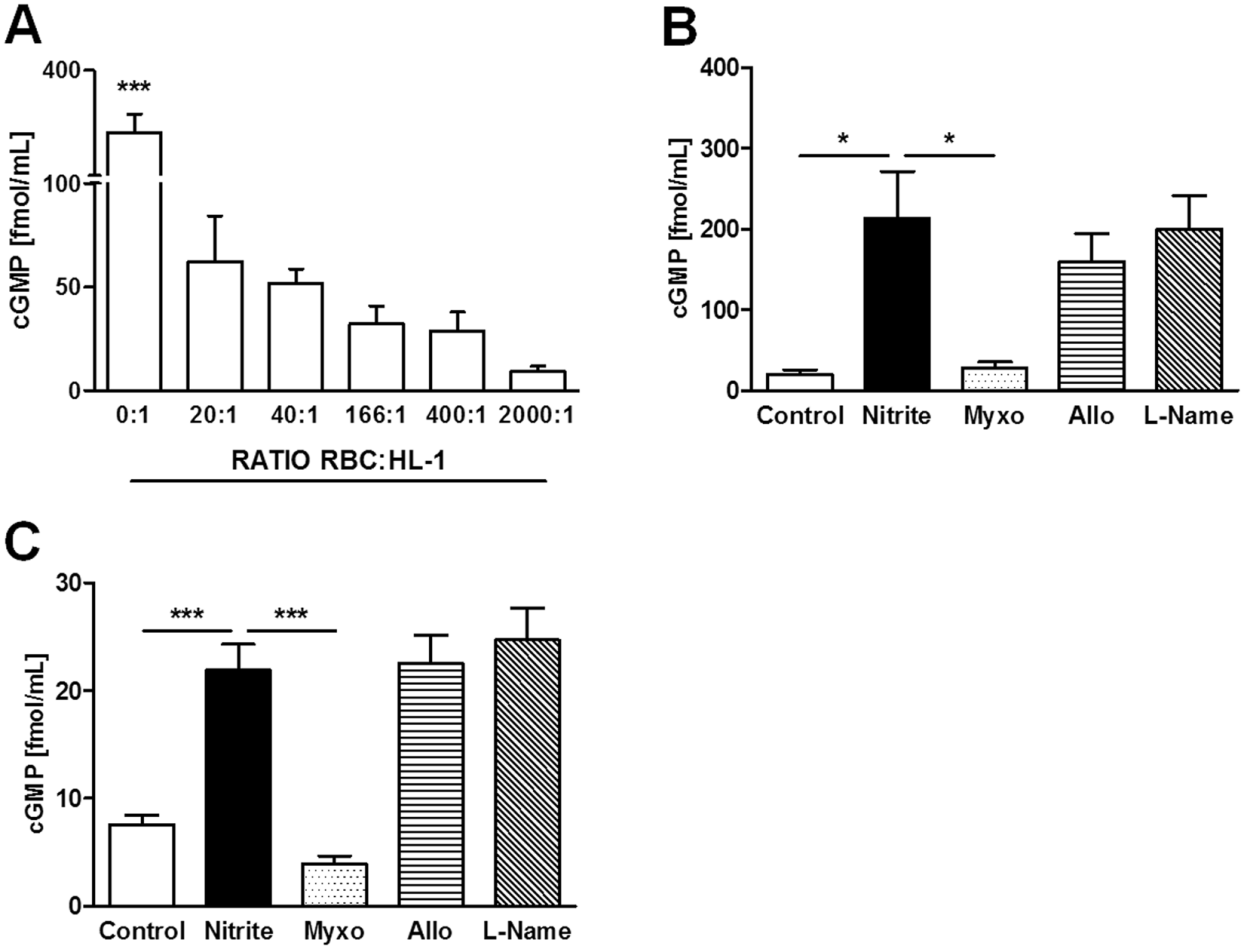
(**A–C**) Intracellular effects contributing to hemodynamic parameters assessed by cGMP synthesis in different cell cultures. Effect of RBCs on nitrite mediated cGMP synthesis on HL1-cardiomyocytes (**A**). Effect of myxothiazol (Myxo), a specific inhibitor of mitochondrial complex III, allopurinol (Allo), a specific inhibitor xanthine oxireductase and N (G)-nitro-L- arginine methyl ester (L-Name), a specific inhibitor of NOS on nitrite mediated cGMP synthesis on HL-1 cardiomyocytes (**B**) and BRL hepatocytes (**C**). Data are expressed as mean ± SEM of at least n = 3, *p < 0.05, ***p < 0.001.

In this model, an increase in the ratio of RBCs to HL-1 resulted in a decrease in cGMP synthesis (Fig. 7A). In addition, we compared the impact nitrite reductases and NOS inhibitors (myxothiazol, allopurinol, L-NAME) on nitrite-mediated cGMP synthesis in cardiomyocytes and hepatocytes (Fig. 7B,C). Hepatocytes were used as a reference cell type. Myxothiazol completely inhibited cGMP synthesis in both cell types as it was shown in previous experiment. In contrast, allopurinol, a specific inhibitor of XOR, and L-NAME a specific inhibitor of NOS, did not have any significant effect on NO release.

## Discussion

In the present study we combined *in vivo* and *in vitro* models to analyze the contribution of single NRs to the regulation of hemodynamics under different oxygen tensions and RBC counts. In the *in vivo* volume replacement model we investigated the impact of NRs located in RBCs (tentatively Hb and XOR) on hemodynamic parameters under normoxic and hypoxic conditions. In subsequent *ex vivo* studies, we dissected the contribution of single NRs located in tissues and conditions required for their optimal function.

Injection of nitrite in rats (i.v.) elevated plasma levels of both nitrite and nitrate up to 40–60 µM after 10 min and dropped down below 5 µM by 20 min after injection (Fig. 2). This was accompanied by a temporary decrease in RI, MAP, PWV and SYS, observed by 2 min and recovered already after 10 min to baseline under normoxic conditions (Hct40-NOX). Under moderate hypoxia (Hct40-HOX; pO_2_ = 58 ± 5 mmHg) PWV, RI, MAP and SYS were also affected temporarily and recovered to baseline by 20 min without displaying any significant difference between Hct40-NOX and Hct40-HOX at any time point. In contrast, heart rate (HR), which was not changed upon nitrite infusion under normoxic conditions, was remarkably decreased when nitrite was infused under hypoxia (Figs 3, 4). A decrease in HR by nitrite is in line with previous publications^25^. Reduction of nitrite to NO by Hb is accompanied by the formation of metHb. However, there were no differences in metHb levels between the groups (Fig. 3C), because metHb is formed at both normoxic and hypoxic conditions by the reaction of nitrite with oxyHb and deoxyHb, respectively^28,29^. In contrast, NO-Hb levels were increased upon hypoxia reflecting increased formation of NO instead of nitrate, (Fig. 3D) facilitating effects of NO derived from RBCs, such as on heart rate. Logically, if the effects of nitrite on the heart rate are mediated by RBCs, then these effects should be diminished by a decrease in RBC counts. In fact, the decrease in Hct abolished the NO-like effect of nitrite on heart rate. Another logic consequence is that RBCs should compete with tissues for nitrite. If so, then a decrease in RBC counts should facilitate non-RBC mediated, tissue-located nitrite pathways. In fact (Fig. 3), a decrease in Hct facilitated several NO-like effects of nitrite such as reducing arterial stiffness (a decrease in PWV), peripheral resistance (RI), mean arterial pressure (MAP), and myocardial contractility (SYS). A concurrence between RBC-dependent and RBC–independent reactions of nitrite can be the logic explanation for this phenomenon, suggesting that RI, MAP, SYS and partially PWV are regulated by NRs located in tissue and RBCs slows down these reactions by intercepting a significant portion of nitrite.

We assume that nitrite-derived NO formed in the blood affects heart rate via interaction of NO with the cardiac conduction system. Purkinje fibers, a part of the cardiac conduction system capable of acting as pacemakers, are located directly beneath the endocardium and are the most probable target for active nitrogen species formed in the blood. It is known, that the interaction of NO with pacemaker cells decreases heart rate by stimulating cAMP breakdown via the upregulation of phosphodiesterase^30^. We assume that NO derived from RBCs can only be active in the heart (and thus can only influence heart rate) because here the ratio RBCs/tissue is very high to generate sufficient amount of NO in blood compartment; This high ratio is necessary, because RBCs are a weak NO generator as our *ex vivo* experiments showed. In contrast, in tissues the ratio RBC/tissue is very low and the amount of NO formed by RBCs in small vessels is not sufficient to influence hemodynamics in tissues (See Suppl. Figure 3). The underlying pathway of NO action might be the interaction with Purkinje fibers. Purkinje fibers are part of the cardiac conduction system capable of acting as pacemakers, which are located directly beneath the endocardium and are the most probable target for active nitrogen species formed in the blood (Suppl. Figure 3).

The next part of our study focused on the oxygen-dependency of NRs located in tissue and blood. We designed *in vitro* experiments to investigate the conditions required for NO release by diverse tissue-located NRs using a chemiluminescence-based NO assay (NOA) combined with a self-designed chamber for on-line detection of NO-release (Suppl. Figure 4). We tested the NO release at three different oxygen levels, namely 0.0 µM O_2_, 3.1 µM O_2_ and 42 µM O_2_. Last two oxygen tensions were selected because they correspond to the concentrations at which Mb and Hb are half saturated with oxygen (P50), respectively. P50 value for Mb is nearly 10-times lower than for Hb (2.8 vs 26 mmHg, respectively; see Suppl. Figure 5). Half saturation of heme is known to provide the optimal nitrite reductase activity^11^. Fully oxygenated hemes do not reduce nitrite, no more turning it into inactive nitrate. Addition of nitrite to RBCs resulted in immediate NO release reaching a maximum approx. 2 min after addition of nitrite (Fig. 4A). The magnitude of this peak correlated with O_2_ concentrations; at 42 µM O_2_ approx. 60% compared to 0.0 O_2_ was present. These data suggest that this peak reflects the reduction of nitrite by Hb because of its coincidence with Hb half saturation.

The fact, that NO release was only transient (peak) and did not reach a constant plateau suggests that NO is able to leave the RBCs and can be detected by NOA, but at the same time also binds to Hb, inhibiting its NR capacity leading to a swift decrease in NO-formation. This very short time of NO release from RBCs may be the reason that it was not observed by others^31,32^ in similar *in vitro* settings. In addition, the chemiluminescence-based technique used here is very sensitive.

A similar but less intensive peak at 2 min was observed in heart homogenate, which most probably was caused by a low amount of blood in the heart tissue homogenate. However, the major peak of NO release in heart homogenates appeared after approx. 12 min. To identify the NR responsible for this peak we tested the NO release at different O_2_ tensions. The increase in O_2_ concentration to 3.1 µM (half-saturation of Mb) did not significantly influence the kinetics of NO-release. When the O_2_ concentration was increased to 42 µM (corresponding to half-saturation of Hb and full saturation of Mb with O_2_) the peak at 12 min nearly disappeared, suggesting that it resulted from the reductase activity of Mb. Instead of this, a weaker peak of NO release occurring by 22 min was observed.

Our assumption that Mb contributes to NO release only at low oxygen tensions is in line with the report demonstrating the abolishment of Mb-related vasorelaxation of aorta rings if the O_2_ level was higher than 1%^33^. To understand the nature of the peak of NO-release observed at 22 min, which occurred at 42 µM O_2_, we examined the effect of myxothiazol, an inhibitor of complex III in mitochondria on the NO generation, in heart homogenates at 0.0 µM O_2_ and 42 µM O_2_. NO release at 0.0 µM O_2_ was only partially inhibited by myxothiazol. More than 70% of the produced NO was not sensitive to myxothiazol and, thus, can be attributed to Mb as assumed above. In contrast, the peak at 22 min which appeared at 42 µM O_2_ was almost completely inhibited by myxothiazol, suggesting that it can be attributed to the mitochondrial respiratory chain. The absolute inhibitory effect of myxothiazol appeared to be very similar for both O_2_ concentrations suggesting that in contrast to Mb and Hb the mitochondrial nitrite reductase is oxygen independent. Indeed, hemodynamic effects of nitrite were already documented in *in vivo* models under physiological pO_2_^23,34^, although they were weaker compared to those observed under hypoxia^23^. Also in this study we observed moderate NO-like effects which appeared under normoxic conditions (Fig. 4).

Since in our *in vivo* experimetns the infusion of nitrite changed myocardial contractility in a RBC-independent manner and in our *in vitro* experiments using heart tissue we demonstrated that NO release from nitrite is catalyzed by both Mb and mitochondria we aimed to understand whether both NR equally contribute to the effects of nitrite or if there is only one dominating. Taking into account that NO-mediated regulation of myocardial contractility is cGMP dependent, we tested the effects of nitrite on NO-generation and cGMP levels in a co-culture model of cardiomyocytes and RBCs (Fig. 6).

Furthermore, the re-distribution of nitrite between RBCs and tissues was assessed *in vitro* using a co-culture model of cardiomyocytes and RBCs in which the released NO was determined by formation of NO-Hb complexes in RBCs as it was done in *in vivo* experiments (Fig. 6). To dissect the contribution of mitochondria in this process, we used myxothiazol, a specific complex III inhibitor of mitochondrial respiratory chain, which completely blocks mitochondrial NR^34^. We observed that myxothiazol and KCN, another mitochondrial inhibitor, only partially inhibited nitrite-mediated NO formation in cardiomyocytes, suggesting that both Mb and mitochondria contribute to this process as it was expected. However, both inhibitors completely abolished cGMP production, showing that although both Mb and mitochondria reduce nitrite to NO, only mitochondrial NO induces cGMP synthesis. The underlying mechanisms of this phenomenon are not clear. Probably NO derived from mitochondria is in closer vicinity to guanylyl cyclase compared to NO formed by Mb or any other intracellular NR.

To identify whether other enzymatic system(s) also contribute to the intracellular nitrite-mediated generation of cGMP, we investigated the effects of additional inhibitors, namely allopurinol, an inhibitor of xanthine oxireductase, and L-NAME, an inhibitor of NOS. In this experiment, we used cardiomyocytes and hepatocytes, the latter used as a reference cell type. Our data showed that only myxothiazol abolished the nitrite-mediated generation of cGMP in both cell types. These data suggest that mitochondria are predominantly responsible for nitrite-mediated cGMP generation.

Finally we tested whether an increase in the number of RBCs would inhibit mitochondrial-mediated cGMP formation due to redistribution of nitrite between RBCs and cardiomyocytes in favor of RBCs as it was suggested by our *in vivo* experiments. Indeed, the generation of cGMP in response to nitrite was gradually inhibited with increasing RBC concentrations (Fig. 7) similarly to *in vivo* data obtained with different Hct values; the nitrite-mediated changes in MAP, RI, PWV and SYS were not seen under normal Hct (Hc40) but only under Hct20.

In this study, we tested allopurinol which is known as an inhibitor of XOR. However, it has been shown that allopurinol in addition to inhibition of XOR also interferes with a number of intracellular signaling cascades such as activation of cytokines response. Recently it has been shown that the induction of cytokines by allopurinol is not related to xanthine oxidase inhibition^35^. Here we show that allopurinol inhibit the reduction of nitrite by Mb, supporting the assumption that allopurinol may influence enzymes other than XOR. These diverse effects of allopurinol can be the reason for the discrepancy of data obtained in tissue homogenates and in cell culture, showing an inhibitory and no effect, respectively. We consider that similar patterns of NO release in heart homogenate and in myoglobin solution suggest the predominant role of myoglobin at very low oxygen tensions and increasing role of mitochondria at the moderate hypoxia.

In summary, we show that under mild hypoxia and normoxia, Hb/RBC-derived NO affects heart rate, while Mb- and mitochondria-derived NO are responsible for other hemodynamic effects of nitrite. Mitochondria-derived NO preferentially regulates myocardial contractility. Mitochondrial NR operates at physiological oxygen partial pressure while Mb is efficient only at very low oxygen tensions. All together, our data suggest that a network of NRs is involved in nitrite mediated regulation of hemodynamics. Oxygen tension and hematocrit define the activity of specific NRs. Our data show that single NRs regulate specific hemodynamic parameters and suggest the application of nitrite for therapeutic purposes for fine tuning hemodynamics.

## Methods

### Experimental design *in vivo*

#### Animals

Male Sprague Dawley rats (n = 27; 390–490 g; Animal Research Laboratories, Himberg) were kept under controlled standard conditions with free access to standard laboratory rodent food and water during an adaptation period of at least 7 days before use in this study. All experimental procedures were in accordance with the Guide for the Care and Use of Laboratory Animals as defined by the National Institutes of Health and were approved by the Animal Protocol Review Board of the city government of Vienna, Austria.

### Anesthesia and instrumentation

Rats were deeply sedated in a pre-flooded box with 3% isoflurane for 1–2 minutes. To maintain surgical depth anesthesia during the instrumentation period, 1% isoflurane was administered via an inhalation mask. The left femoral vein was cannulated with a silicone catheter (0.025″ × 0.047″ medical grade tubing, Degania Silicone Ltd., Israel) and connected to a three-way stopcock for intravenous (i.v.) application of anesthetics and fluid treatment. After disconnection of isoflurane, anesthesia was continued by intravenous (i.v.) application of S-ketamine at a rate of 66 mg/kg/hr in combination with xylazine (2.5 mg/kg intramuscularly). Both femoral arteries were cannulated with a 24 G catheter (Vasofix Certo, Luer Lock, B.Braun Melsungen) and connected to a three-way stopcock for measurement of hemodynamic parameters and withdrawal of blood. The animals were positioned on a temperature-controlled surgical board (36–37 °C) during the entire experiment.

### Experimental set up

Animals were randomly assigned to three different groups of nine animals each: (a) with normal hematocrit (Hct) under normoxic conditions (Hct40-NOX), (b) with normal Hct under hypoxic conditions (Hct40-HOX) and (c) halved Hct under hypoxic conditions (Hct20-HOX). The experimental procedure is illustrated in Fig. 1A. After instrumentation of the vessels, animals were allowed to stabilize for at least five minutes before baseline sampling. Hematocrit adjustment (HCT ADJ) was initiated by infusion of Ringer’s solution (1.5 mL/min) and simultaneously pump-driven withdrawal of blood from the right femoral artery (0.5 mL/min) for the duration of ten minutes. The procedure was continued until a Hct of 20 ± 2% was reached. After HCT ADJ arterial oxygen pressure (pO_2_) was adjusted (NOX/HOX). Hypoxia was induced by inhalation of nitrogen/air mixture containing ca. 15% oxygen (O_2_). The O_2_ concentration was adjusted to keep pO_2_ at 50 mmHg. After ten minutes of either normoxia (room air, 21% O_2_) or hypoxia, animals received an i.v. bolus of nitrite (15 µmol/kg). Blood pressure, heart rate and pulse waves were continuously monitored during the entire time of the experiment and analyzed before nitrite infusion (BN), 2, 10, and 20 minutes after nitrite infusion using PowerLab software system (ADInstruments, Hungary). Whole blood samples were taken at baseline (BL), after HCT ADJ, ten minutes after pO_2_ adjustment (NOX/HOX), 10 and 20 minutes after nitrite administration.

### Blood sampling and analysis

For blood gas analysis, 0.3 mL heparinized arterial blood samples were taken and analyzed with a blood gas analyzer ABL 625 System (Radiometer Medical A/S, Copenhagen, Denmark). For nitric oxide hemoglobin (NO-Hb) and cyclic guanosine monophosphate (cGMP) analysis 0.5 mL EDTA blood samples were taken. Of these 0.5 mL a 150 µL aliquot of whole blood were transferred into a plastic pipette and immediately frozen in liquid nitrogen and kept at −80 °C for NO-Hb analysis by electron paramagnetic resonance (EPR) analysis. The remaining blood was centrifuged (Centrifuge 5415 R, Eppendorf, Germany) at 7200 rpm for 10 minutes at 4 °C and the plasma supernatant stored at −80 °C for cGMP analysis.

### Contour analysis of pulse (pressure) waves

Pulse wave analysis (also called pressure wave analysis) was performed as previously described^36–39^. We analysed the contour of the digital pressure pulse via a catheter placed in the v. Femoralis. The contour of pulse waves observed in our study was similar to those described previously^39,40^. The waves consisted of two peaks. The first peak is the incidental pressure wave generated from the heart while the second peak is generated from the reflection of the incidental wave from the reflection site of the peripheral part of the body. The peak-to-peak time (indicated as the pulse/pressure wave velocity, PWV) represents the transit time between the incidental and reflective waves. Using this parameter, the stiffness index (SI) can be calculated by dividing height of the subject by the PWV^37,39^, because the travel distance of the reflective wave is proportional to the height (h) of the subject. In our case, the animals had the same size and we considered the ratio 1/PWV to estimate SI. This parameter characterizes the arterial stiffness in the femoral segment of arterial tree. Another commonly used parameter is reflective index (RI), calculated as the ratio of the height of the reflective wave (b) to the incidental wave (a). Arterial stiffness and reflection index were determined as previously described in references cited above, particularly detailed described in^39^. More recently another parameter, the maximum slope of the vascular pulse wave (dP/dt) during the upstroke was suggested as the measure of cardiac contraction force^41^. This parameter was also determined in our study in order to estimate contractility of myocardium. The major advantage of this method is that it provides several parameters of interest with a minimal invasion, but the parameters listed above are relative, and therefore must be considered individually. Therefore, to dissect the effects of nitrite on hemodynamic parameters, we always waited until the changes induced by pre-treatments (anesthesia, hypoxia, hematocrit etc.) reached a steady state, then nitrite was infused and the changes induced by nitrite were normalized to the steady state value for each animal individually.

In our study the pulse waves were recorded using a PowerLab software system (ADInstrument, Budapest, Hungary). To determine the hemodynamic changes in response to nitrite, pulse wave analysis was performed (Fig. 3A). For precise definition, the first derivative plot was used for the diastolic component of the pulse wave (dotted line) and systolic slope (SYS, characterizing myocardial contractility). Heart rate (HR), mean arterial pressure (MAP), pulse wave velocity (PWV, characterizing aorta stiffness), reflection index (RI, characterising peripheral vascular resistance), and SYS were analyzed.

### Experimental design *in vitro*

#### Cell culture

Cell cultures of HL-1 cardiomyocytes and BRL-3A hepatocytes were used in this study.

HL-1 cardiomyocytes and hepatocytes were cultured under 5% CO_2_ atmosphere in Claycomb medium (Sigma, St. Louis, USA) supplemented with 10% fetal bovine serum (Lonza, Basel, Switzerland), 4 mM L-glutamine (Sigma, St. Louis, USA), 100 U/mL penicillin (Sigma, St. Louis, USA), 100 µg/mL streptomycin (Sigma, St. Louis, USA), and 100 µM norepinephrine (Sigma, St. Louis, USA). Cells were seeded in 24-well cell culture plates pre-coated with 25 µg/mL fibronectin and 0.02% gelatine solution and cultivated until confluence.

### Experimental set up

Experiments were performed in a gas-tight glove box under nitrogen atmosphere (at < 1% O_2_). All solutions were deoxygenated with nitrogen prior to experiment. To isolate human RBCs, whole blood was centrifuged at 1600 g for 10 min. The plasma and buffy-coat were discarded and the remaining RBCs were washed twice with phosphate buffered saline (PBS). RBCs were then diluted with PBS accordingly, counted on a cell counter Cell-Dyn 3700 (Abbott, Switzerland) and deoxygenated with nitrogen. 24-well cell culture plates with confluent cells (2.5 × 10^5^ cells/well) were transferred into the glove box where the cell medium was exchanged with diluted red blood cells or PBS as a control, followed by addition of 50 µM nitrite. Cells were then incubated under hypoxic conditions on a shaker at 37 °C for 1 hour, after which the supernatant was drawn in syringes, shock-frozen in liquid nitrogen and stored for EPR analysis of NO-Hb complexes at −80 °C. In a number of experiments cells were pre-incubated for 1 hour with either 10 mM potassium cyanide (KCN), 10 µM myxothiazol (Myxo), 1 mM allopurinol (Allo) or 300 µM N-nitro-L-arginine methyl ester (L-NAME), respectively.

### cGMP analysis

Under nitrogen atmosphere, medium was replaced by PBS containing 1 mM of the phosphodiesterase inhibitor isobutylmethylxanthin (IBMX) to prevent cGMP degradation. After nitrite addition and 1 hour incubation the supernatant was removed and the adherent cells lysed by sonification in 500 µL lysis reagent provided with the enzyme immunoassay for detection of cGMP (cGMP EIA, GE Healthcare, Fairfield, USA). Protein content of the lysates was determined by BCA protein assay (Pierce, Rockford, USA). The cell lysate was used to analyse cGMP. cGMP levels were measured in acetylated samples according to protocol 3 of the manufacturer’s instructions. Optical density (405 nm) was measured by a plate reader (Tecan, Männedorf, Switzerland) and the concentration of cGMP was calculated from a standard curve produced from serial dilutions of acetylated cGMP solutions. All standards and samples were measured in duplicates.

### Analysis of nitrite and total NO (NOx)

Nitrite and total NO in plasma were measured with a Sievers 280i NO Analyzer (Sievers, Boulder, USA) in two separate runs. Plasma samples were injected through a septum into the glass vessel, where NO species were converted by a redox active reagent (NaI for nitrite and VCl_3_ for total NO analysis, respectively) to NO_(g)_, A subsequent reaction with O_3_ caused photon emission, which was detected as chemiluminescence.

### Electron paramagnetic resonance (EPR) spectroscopy

As a metabolic product of nitric oxide (NO), NO-Hb was determined in frozen blood samples using EPR spectroscopy. The spectra were recorded at liquid nitrogen temperature using an X-band EPR spectrometer (MiniScope MS200, Magnatech, Berlin, Germany) using the following settings: microwave frequency 9.429 GHz, microwave power 30 mW, modulation frequency 100 kHz, modulation amplitude 6 G.

### Determination of NO release

NO release was determined using a nitric oxide analyser (Sievers 280i, General Electrics, USA) which was connected to a specially designed glass chamber that allows the use of large sample volumes (up to 100 mL) and substantially increased the sensitivity of the method (Suppl. Figure 3). Gas mixtures of different oxygen concentrations were prepared by mixing medical grade air or oxygen with gaseous nitrogen (99.99% grade). The measurements were carried out using RBC suspensions, heart homogenates obtained from rats and myoglobin from horse heart (Sigma, Austria). Samples were prepared immediately before the experiment. For RBC isolation, approx. 12 mL of rat blood were diluted with NaCl solution (0.9%) and centrifuged (Megafuge 1.0 R, Haerus Instruments, Germany) at 3000 rpm, 4 °C. After removing the supernatant the washing step was repeated and the remaining RBCs were kept on ice until measurement. For the preparation of heart homogenates, muscle tissue of two rat hearts was pooled and divided into 3 aliquots of at least 0.7 g. Subsequently, one aliquot was homogenized at a ratio of 1:32.5 (w/v) in buffer (pH 7.4) containing 80 mM KCl, 5 mM KH_2_PO_4_, 20 mM Tris-HCl and 1 mM DETAPAC (Sigma, Austria) and kept on ice until measurement. Prior to the myoglobin measurements, a solution of 35 µM myoglobin in buffer was deoxygenated with sodium dithionite salt, which was then removed by means of a dialysis cassette (7 k molecular weight cut-off, Thermo Scientific). After sample preparation, 16.5 mL were transferred into the glass chamber and equilibrated at one of the following O_2_ concentrations: 0.0 µM, 3.1 µM, and 42 µM. Following this, nitrite solution was immediately added to a final concentration of 15.6 mM. This concentration of nitrite enables stable on-line measurements of NO production for up to 45 minutes. The NO release from nitrite was measured over a time course of at least 20 minutes. For the determination of individual contributions of nitrite reductases in heart homogenates, 150 µM of the xanthine oxidoreductase inhibitor Allopurinol (Sigma, Austira) was added either prior to the measurement or during the plateau phase in NO release after 15 minutes. In order to assess the stability of the measuring system in the presence of different gas mixtures, different amounts of NO-gas dissolved in water serving as positive controls were injected into the chamber according to the previous mentioned protocol.

### Statistical analysis and data presentation

Statistical analysis was performed by repeated measures two-way ANOVA followed by Bonferroni post hoc test for the three treatment groups. One-way ANOVA followed by Bonforroni post hoc test was performed for cGMP analysis. The number of animals and independent samples (n) are indicated in figure legends. Data are represented as mean ± SEM. The significance level was set at 0.05 and is indicated as *p < 0.05, **p < 0.01, ***p < 0,001. Calculations were performed using GraphPad Prism 5.01 for Windows, GraphPad Software, San Diego California USA.

## Electronic supplementary material

Supplementary information
