# Supplementary material for: Impact of mitochondrial nitrite reductase on hemodynamics and myocardial contractility
Source: Sci Rep. 2017 Sep 21;7:12092. doi: 10.1038/s41598-017-11531-3 (PMC5608763; doi:10.1038/s41598-017-11531-3)
Supplement: Supplementary file 1 — Supplementary information [file 41598_2017_11531_MOESM1_ESM.pdf]

Supplementary information

Impact of mitochondrial nitrite reductase on hemodynamics and myocardial contractility

*Peter Dungel(PhD), *Carina Penzenstadler(DVM), Mostafa Ashmwe, Sergiu Dumitrescu(MSc), Tanja Stoegerer, Heinz Redl(PhD), Soheyl Bahrami(PhD), Andrey V Kozlov(MD, PhD).

L. Boltzmann Institute for Experimental and Clinical Traumatology in AUVA center, Vienna, Austria

Donaueschingenstraße 13; 1200 Vienna, Austria;

Tel: +43-059393-41980;

Fax: +43-059393-41982;

Corresponding author: Andrey V Kozlov

E-mail: andrey.kozlov@trauma.lbg.ac.at

* Equal contribution

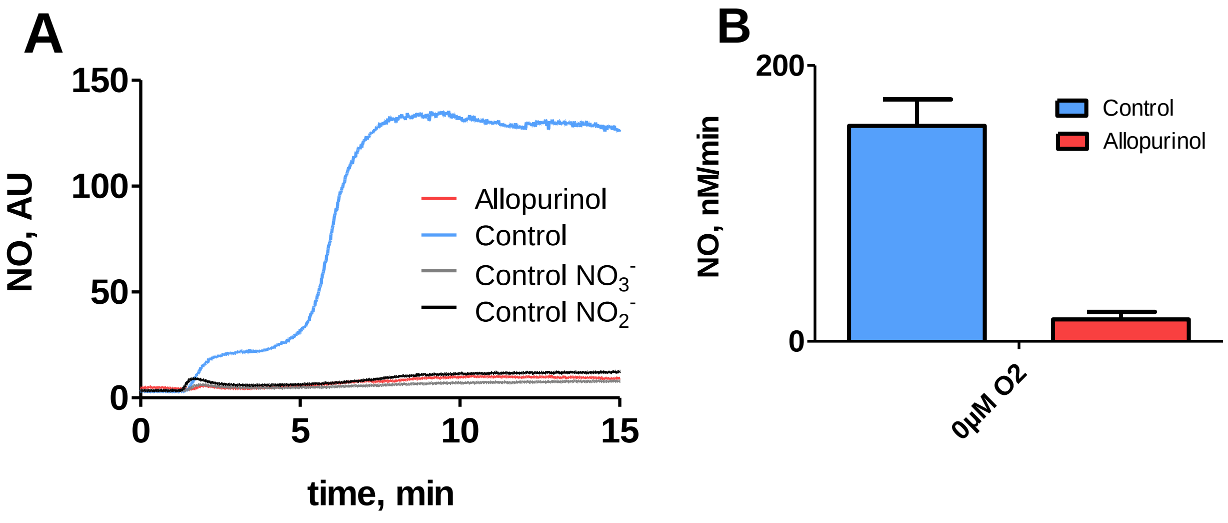

Suppl. Figure 1. The release of NO from nitrite, nitrate and nitrite incubated with heart homogenate in the presence and absence of allopurinol. A- kinetics; B – statistical evaluation.

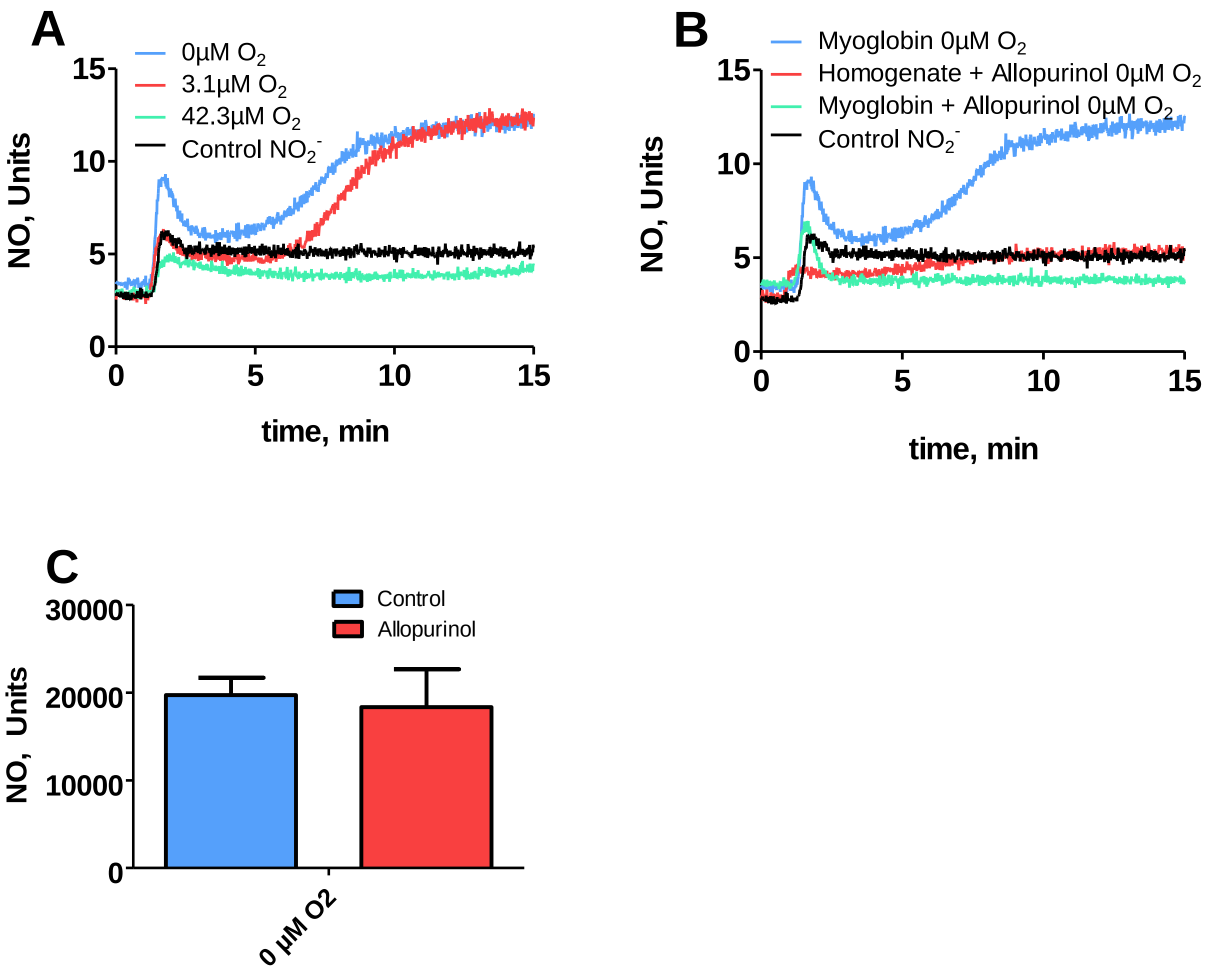

Suppl. Figure 2. Effect of oxygen and allopurinol on the Mb catalyzed release of NO from nitrite and effect of allopurinol on the NO concentration in a saline solution under anaerobic condition. A – Effect of oxygen concentrations; B – effect of allopurinol; C – effect of allopurinol on NO levels in aqueous solution.

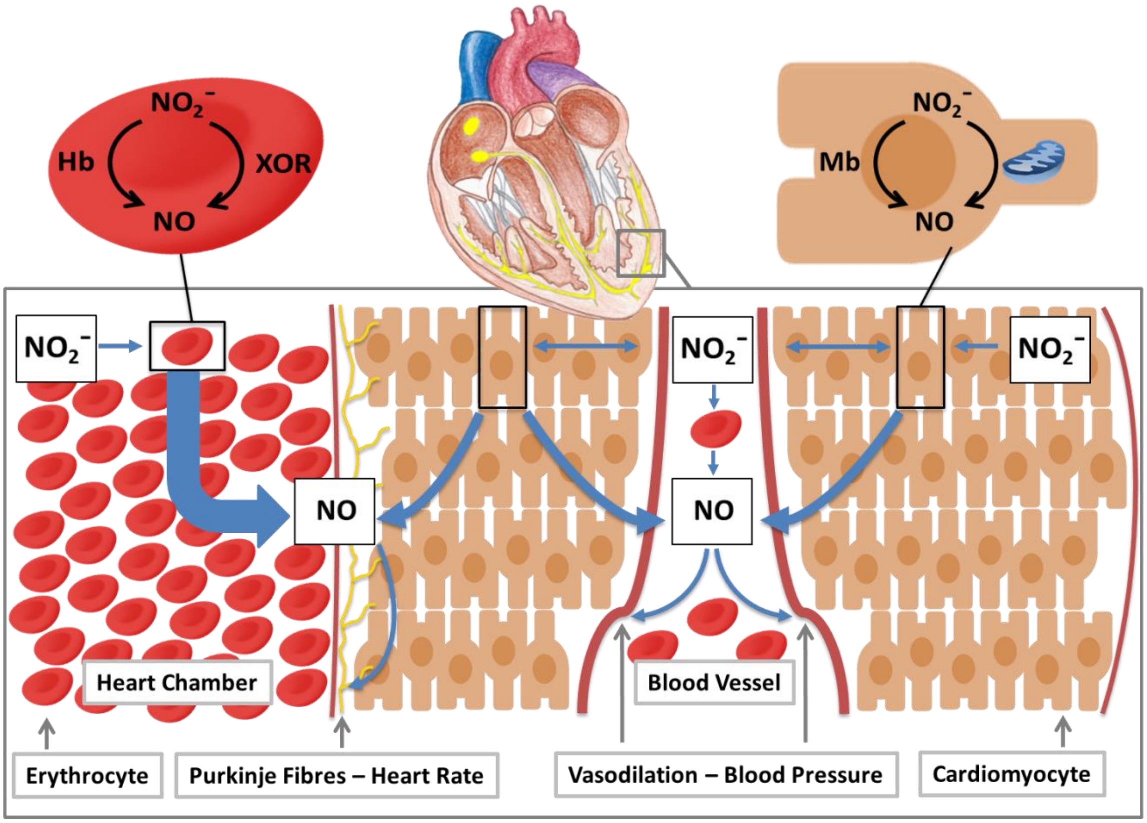

Suppl. Figure 3. Hypothetic scheme illustrating regulation of hemodynamics by the nitrite reductases. Nitrite (NO2-) is converted to nitric oxide (NO) in the heart (area depicted in grey box) in the following pathways. In erythrocytes, which are lacking of mitochondria, nitrite is reduced to NO via hemoglobin (Hb) or/and xanthin oxireductase (XOR) pathways. NO formed in blood from nitrite mainly controls the heart rate by interacting with the Purkinje fibers, which are directly located beneath the endocardium and therefore the most probable target for active nitrogen species. NO reduced from nitrite is additionally required for the regulation of blood pressure. In cardiac tissue (also likely in other tissues), nitrite is reduced to NO, via myoglobin (Mb) or/and mitochondria pathways. NO formed in cardiac tissue regulates the peripheral resistance, blood pressure and myocardial contractility.

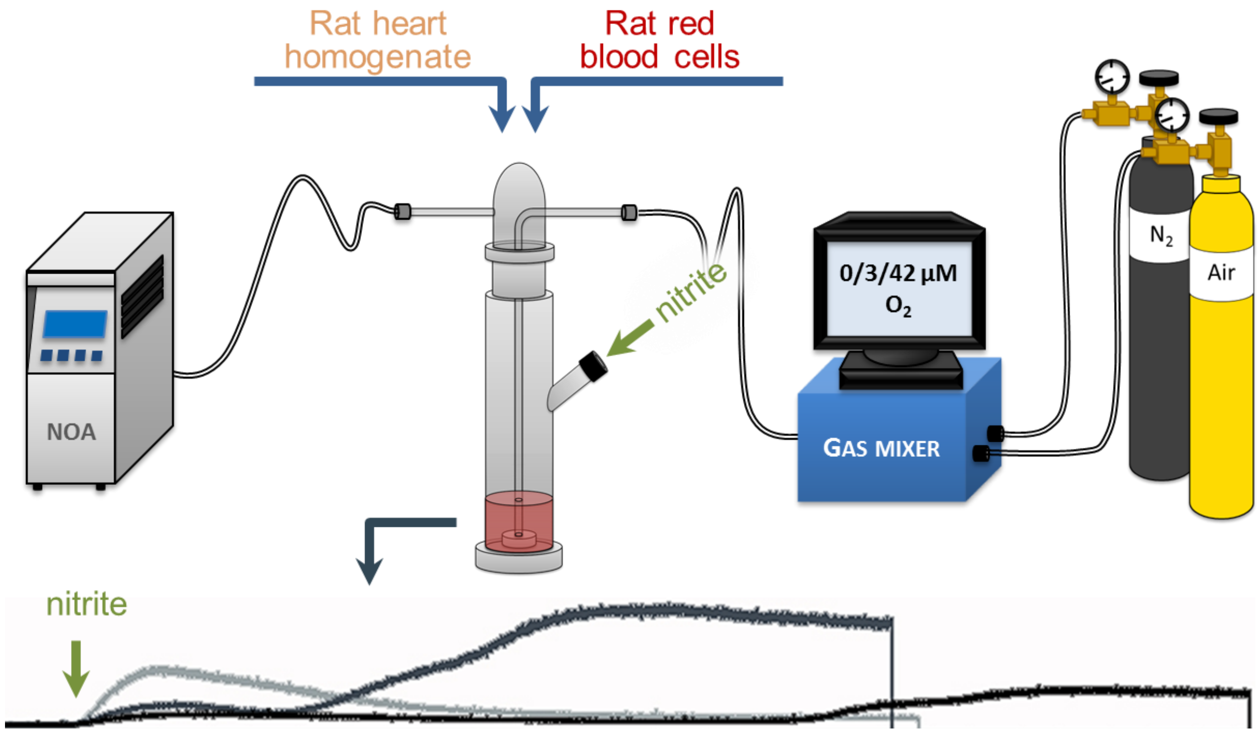

Suppl. Figure 4. Schematic illustration of the experimental set-up to determine nitrite-derived NO release under various O2 concentrations. The setup was based on a chemoluminescent nitric oxide analyser (Sievers 280i, General Electrics, USA) using a specially designed glass chamber. The gas inlet was connected to a gas mixing device to compose gas mixtures with different oxygen contents. The measurements were carried out with RBC suspensions and heart homogenates from rats. The NO release from nitrite was measured over a time course of at least 20 minutes. In order to assess the stability of the measuring system in the presence of different gas mixtures, different amounts of NO-gas dissolved in water serving as positive controls were injected into the chamber.

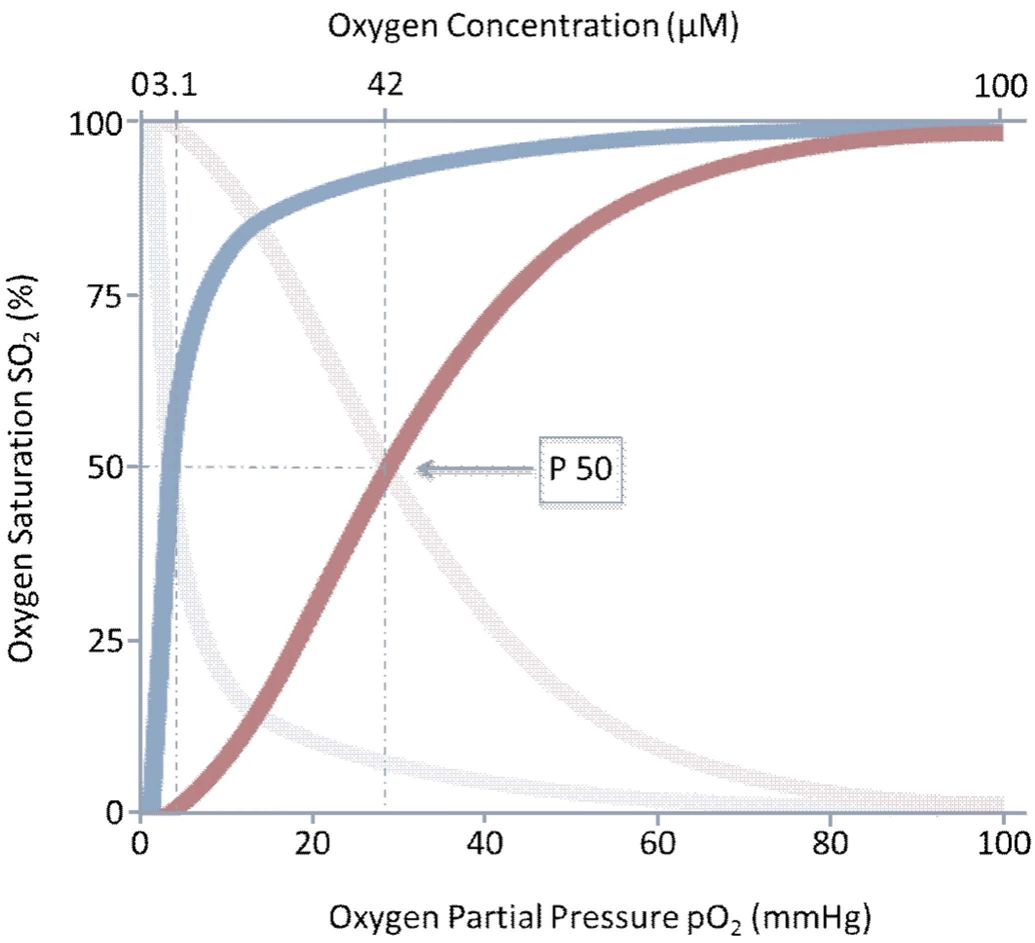

Suppl.Figure 5. Oxygen dissociation curves of oxygenated (dark coloured) and deoxygenated (light coloured) hemoglobin (red) and myoglobin (blue). The P50 term indicates the partial pressure of oxygen at which the oxygen carrying protein (Hb in red, Mb in blue) is 50% saturated. The upper x-axis refers to the oxygen concentrations which several nitrite reductases are active (0.0 µM - all nitrite reductases are active; 3.1 µM – Hb and Mb are active; 42 µM – only Hb is active).
